# Reconstitution of Cholesterol-Dependent Vaginolysin into Tethered Phospholipid Bilayers: Implications for Bioanalysis

**DOI:** 10.1371/journal.pone.0082536

**Published:** 2013-12-13

**Authors:** Rima Budvytyte, Milda Pleckaityte, Aurelija Zvirbliene, David J. Vanderah, Gintaras Valincius

**Affiliations:** 1 Department of Bioelectrochemistry and Biospectroscopy, Institute of Biochemistry, Vilnius University, Vilnius, Lithuania; 2 Department of Immunology and Cell Biology, Institute of Biotechnology, Vilnius University, Vilnius, Lithuania; 3 Biomolecular Structure and Function Group, National Institute of Standards and Technology at Institute of Bioscience and Biotechnology Research, Rockville, Maryland, United States of America; 4 Bio Complexity Department, The Niels Bohr Institute, Copenhagen University, Copenhagen, Denmark; Duke University Medical Center, United States of America

## Abstract

Functional reconstitution of the cholesterol-dependent cytolysin vaginolysin (VLY) from *Gardnerella vaginalis* into artificial tethered bilayer membranes (tBLMs) has been accomplished. The reconstitution of VLY was followed in real-time by electrochemical impedance spectroscopy (EIS). Changes of the EIS parameters of the tBLMs upon exposure to VLY solutions were consistent with the formation of water-filled pores in the membranes. It was found that reconstitution of VLY is a strictly cholesterol-dependent, irreversible process. At a constant cholesterol concentration reconstitution of VLY occurred in a concentration-dependent manner, thus allowing the monitoring of VLY concentration and activity *in vitro* and opening possibilities for tBLM utilization in bioanalysis. EIS methodology allowed us to detect VLY down to 0.5 nM (28 ng/mL) concentration. Inactivation of VLY by certain amino acid substitutions led to noticeably lesser tBLM damage. Pre-incubation of VLY with the neutralizing monoclonal antibody 9B4 inactivated the VLY membrane damage in a concentration-dependent manner, while the non-neutralizing antibody 21A5 exhibited no effect. These findings demonstrate the biological relevance of the interaction between VLY and the tBLM. The membrane-damaging interaction between VLY and tBLM was observed in the absence of the human CD59 receptor, known to strongly facilitate the hemolytic activity of VLY. Taken together, our study demonstrates the applicability of tBLMs as a bioanalytical platform for the detection of the activity of VLY and possibly other cholesterol-dependent cytolysins.

## Introduction

Cholesterol-dependent cytolysins (CDCs) comprise a class of structurally related bacterial pore-forming toxins. CDCs are produced by many gram-positive pathogens [1] and have been considered as virulence factors of bacteria contributing to bacterial invasion and infection [2-4]. In addition, CDCs have been recently identified in non-pathogenic gram-negative species [5]. Vaginolysin (VLY), the toxin of CDC family, is secreted by *Gardnerella vaginalis* [6]. *G. vaginalis* has been identified as the prevailing inhabitant of the vaginal tract of women diagnosed with bacterial vaginosis (BV) [7,8]. BV, a disease characterized by malodorous vaginal discharge, is linked with infertility, adverse pregnancy outcomes, post-surgery infections and may increase the risk of acquiring sexually transmitted diseases [8,9]. Despite a strong correlation between the abundance of *G. vaginalis* and the BV state, the role of *G. vaginalis* was considered elusive [10]. However, recent findings on a link between a structured polymicrobial *G. vaginalis* biofilm covering the endometrium and fetal loss [11] is compelling evidence for the active role of *G. vaginalis* in the degradation of the vaginal mucus [12] and the significance of this particular bacterium in BV. VLY is now considered as a well-recognized virulence factor of *G. vaginalis* [6,13]. In addition, recent data have demonstrated that differing VLY production levels between *G. vaginalis* strains may correlate with the phenotypes of BV [14]. Consequently, fast analytical detection of VLY and its activity, is an important issue in the assessment of the nature of the infection and could facilitate the improvement in existing methods of BV diagnosis. 

Commonly, CDCs activity is determined by *in vitro* assays using either red blood cells or cell lines such as HeLa [6,13]. Alternatively, the amount of CDCs produced by bacteria can be determined by immunoassays if the appropriate antibodies are available [13-16].

We aim at developing an alternative bioanalytical technique that would significantly simplify and speed up the measurement of the activity of the toxin so that analysis may be performed within several minutes. In our approach, we utilize the property of VLY as a member of a CDC group of toxins to bind to cholesterol-containing membranes of target cells. CDC binding leads to pore-formation that triggers cell lysis and death. The formation of defects or water-filled pores in artificial tethered bilayer membranes (tBLMs) [17,18] can be easily sensed and followed, in real-time, by electrochemical techniques, in particular, by electrochemical impedance spectroscopy (EIS) [19-21] and opens the possibility of tBLM use in bioanalytical applications.

Recently, tBLMs in combination with EIS data was applied to the detection of α-hemolysin (αHL) in protein solutions [20] and cell cultures [22,23]. Reconstitution of αHL into phospholipid bilayers has no strict requirement for cholesterol occurring via direct interaction of the protein monomer with the membrane followed by subsequent oligomerization into a heptameric pore [24].

The detailed mechanism of VLY binding is still unknown. It was shown that VLY as well as intermedilysin from *Streptococcus intermedius* and lectinolysin from *Streptococcus mitis* use human CD59 as their receptor rather than cholesterol to bind to a membrane [6,25,26]. CD59 is a glycosyl-phosphatidylinositol (GPI)-anchored membrane protein that blocks the formation of the complement membrane attack complex (MAC) by binding complement proteins C8α and C9 [27]. It is presumed that the requirement of CD59 in membrane binding results in the specificity of VLY to human cells as mouse erythrocytes were 200-fold less susceptible to its hemolytic activity [6,13]. Even though the role of CD59 receptor in VLY toxicity is well-established, it is not yet clear if VLY is capable of producing membrane pores in the absence of this receptor. The strict requirement for both CD59 and cholesterol would make artificial bilayer-based bioanalytical systems more complex and possibly less attractive from a practical point of view. In distinct contrast, the possibility of detecting VLY activity on already well-characterized tBLMs containing no CD59 could lead to the development of fast bioanalytical systems [28].

The objective of this paper was to investigate the reconstitution of VLY in the absence of CD59 into tBLMs and verify their applicability in sensing the bioactivity of CDCs. Dioleoylphosphocholine tBLMs with variable amount of cholesterol have been described and characterized earlier [29]. In the current study we demonstrate that both recombinant and pathogen-produced VLY, but not inactivated VLY mutants specifically inflict dielectric damage to tBLMs, thus opening a gateway for the development of tBLM-based bioanalytical systems for the detection of VLY activity.

## Materials and Methods

### Gold-coated glass slides

Gold-coated glass slides for tBLMs were made in a PVD75 (Lesker, UK) vacuum system using 50.8 mm metal sputtering targets. Typically 50±5 nm thick gold films, deposited atop a previously sputtered layer of Cr (2 nm) to ensure adhesion of the gold film to the glass substrate, were used throughout the work. Magnetron deposition parameters for gold were as follows: sputtering voltage ≈350 V, sputtering pressure of an argon ≈5 mbar; deposition rate ≈0.4 nm/s. High purity (99.9999 %) argon was used as sputtering gas. Before coating, the glass slides were cleaned by i) rinsing with Micro 80 ® solution (Sigma-Aldrich, Germany) and pure water (Milli-Q Plus, USA) , and ii) chemically cleaning in Nochromix ® solution (Sigma-Aldrich, Germany) for 20 min. Both steps were followed by rinsing with copious amount of pure water, filtered by Millex-FH® filter with 0.45 μm pores (Millipore, USA), blow-dried in a stream of 99.99% nitrogen (AGA, Sweden), and immediately transferred into the vacuum chamber. Glass slides (25x75 mm) were from various vendors, including Fischer Scientific.

### Preparation of tethered bilayer membranes

Tethered (phospho) lipid bilayer membranes (tBLMs) were prepared on the gold-coated glass slides by the solvent exchange method [17,30] as described earlier [28]. Briefly, the freshly gold-coated glass slides were immersed overnight, typically 12-18 h, in a 0.2 mM solution of the tether WC14 (20-tetradecyloxy-3,6,9,12,15,18,22-heptaoxahexatricontane-1-thiol, synthesized in-house), and β-mercaptoethanol (Sigma-Aldrich, St.Louis, MO) mixed at molar ratio 3:7 in ethanol (96.3 vol.% ) to form mixed self-assembled monolayers (SAMs). After incubation, the slides were washed with 20-30 ml of pure ethanol (96.3%) and dried in a nitrogen stream. After the formation of the tethering SAMs, the slides were used immediately. However, we established that they could be stored for up to 10 days without losing their ability to form tBLMs. The tether-functionalized gold films were assembled into an electrochemical setup with 6 independent 250-300 µL vials. The bottom of each vial was a patch of the tether-functionalized gold layer and is exposed to the subsequent solutions. The picture of the setup used in this work can be found in the material of ref [29].

.The tBLMs were formed by putting 50 µL of an ethanolic phospholipid solution into the vial, and then rapidly exchanging it (using a syringe) with 15-20 mL of aqueous phosphate buffer (0.1 M NaCl, 0.01 NaH_2_PO_4_ adjusted to pH=7.2±0.1 with NaOH). Purified Milli-Q Plius (Millipore, USA) water was used for all buffers. The membranes were equilibrated with the working phosphate buffer for at least 30 min and only those tBLM samples exhibiting stable parameters were used. Because water-soluble ethanol was used in the preparation of the tBLMs, any residual amount of ethanol in the membranes is reduced to undetectable levels by copious flushing the measurement cell with buffer. The phospholipids – 1,2-dioleoyl-*sn*-glycero-3-phosphocholine (DOPC) and 1,2-diphytanoyl-*sn*-glycero-3-phosphocholine (DPhyPC) – as well as the cholesterol were all purchased from Avanti Polar Lipids (Alabaster, AL) in powder form and used as received. 

### Electrochemical impedance measurements

Electrochemical impedance was measured using a universal electrochemical workstation Zennium (Zahner, Kronach, Germany) in the frequency range between 0.1 Hz and 100 kHz, with 10 logarithmically distributed measurement points per decade. The working surface area exposed to the solution was 0.32 cm^2^. The EIS data is presented normalized to the geometric surface area as calculated from the roughness factor of the magnetron sputtered gold films – estimated from the oxidation/oxide stripping charge of the gold to vary from 1.35 to 1.40. A saturated silver-silver chloride (Ag/AgCl/NaCl (aq. sat.)) microelectrode (M-401F, Microelectrodes, Bedford, NH) was used as reference, which has the potential +196 mV vs. standard hydrogen electrode. All potentials are referred vs. the reference electrode. The auxiliary electrode was a platinum wire (99.99% purity, Aldrich; diameter = 0.25 mm) coiled around the barrel of the reference electrode. Measurements were carried out with 10 mV alternating current at 0 V bias versus the reference electrode in aerated solutions. The admittance of the tBLMs was determined from the electrochemical impedance spectra (see Figure S2 in File S1 for details). Average admittance values and standard errors were obtained from at least 3 independent measurements, on different tBLMs.

### Preparation of liposomes

Vesicles were prepared using 0.001 mol/L chloroform solutions of cholesterol/DOPC at a molar ratio of 40/60, which approximately mimics biological membrane compositions. A lipid film was prepared by evaporating 1 mL of the chloroform solution in a gentle stream of nitrogen followed by vacuum-drying for 1-2 h. The lipid film was re-dissolved in 1 mL of pentane and dried overnight. The film was hydrated by adding 1 mL of working buffer, 0.1 mol/L NaCl, 0.5 x 10^-3^ mol/L NaH_2_PO_4_ (pH 7.4), sonicated for 60 min, and incubated with occasional vortexing, as needed until the lipid film was no longer visible. The lipid preparation was then extruded 21 times through a 100 nm polycarbonate membrane (Avanti Polar Lipids, Alabaster, AL). Vesicle size (≈50-100 nm) was determined by dynamic light scattering as described earlier [31].

### Generation of recombinant VLY

Recombinant N-terminally-hexahistidine-tagged VLY (rVLY) lacking the putative signal sequence [amino acids (aa) 1-31] was expressed and purified as described previously [13].

### Generation of rVLY mutants

All rVLY mutants were generated using pUC57 plasmid carrying the VLY coding gene lacking 1-31 aa as the template for PCR-mediated, site-directed mutagenesis targeted to the whole plasmid. All PCR generated VLY-encoding sequences were confirmed by DNA sequence analysis. Three aa substitutions (A162V; R163V; A162E) targeted the motif located in β1-strand of domain 3. The double mutant (T474G•L475G) was generated according to Farrand et al. [32]. The VLY mutants were cloned into pET28a(+) vector (Merck, Darmstadt, Germany) for construction of hexahistidine-tagged proteins, expressed and purified as described previously [13].

### Circular dichroism (CD)

CD spectra were recorded on a J-815 CD Spectrometer (Jasco) at 25°C over the wavelength interval 190-300 nm at a scan speed of 50 nm/min and averaged from three scans with all protein concentrations at 0.2 mg/mL in 20 mM sodium acetate buffer (pH 5.5). A reference spectrum of the sodium acetate buffer only was recorded and subtracted from the sample spectra. The CD of the rVLY and its mutants were expressed as the mean of the residue ellipticity (MRE). 

### Determination of rVLY hemolytic activity

Hemolytic activity of each rVLY mutant and rVLY was determined on human erythrocytes as described previously [13]. The HD_50_ was defined as the concentration of rVLY required to lyse 50% of human erythrocytes and was determined by hemolytic assay performed in triplicate.

### Supernatants of *G. vaginalis* cultures

The supernatants of *G. vaginalis* strains (GV32, GV33, GV34, GV35 and GV36) used in this study were described previously [14]. The VLY secretion level determined by a sandwich enzyme-linked immunoassay (ELISA) of strains GV32 and GV36 was considered as moderate (0.4-0.8 µg/mL), strains GV33 and GV34 produced low levels of VLY (<0.4 µg/mL) and the level of VLY in the culture of GV35 was not-detectable [14].

### Monoclonal antibodies

The monoclonal antibodies (MAbs) against rVLY used in this study were described previously [13]. For the inhibition of rVLY activity, MAb 9B4 with the most potent neutralizing activity (IC_50_ = 6.7x10^-11^) and MAb 10A6 with a moderate neutralizing activity (IC_50_ = 3.6x10^-9^) were employed. As a negative control for rVLY inhibition experiments, the non-neutralizing MAb 21A5 against rVLY and an irrelevant MAb of IgG1 subtype against measles nucleocapsid protein (clone 22G2) were used [33].

### Enzyme-linked immunosorbent assay (ELISA)

The reactivities of the MAbs with mutant forms of VLY were tested by an indirect ELISA as reported previously for rVLY [13]. Briefly, microtiter plates were coated with purified mutant rVLY by adding 100 μl of a rVLY solution (5 μg/mL) to a coating buffer (50 mM Na_2_CO_3_, pH 9.5) solution and incubated overnight at +4°C. The plates were blocked with 1% bovine serum albumin (BSA) in phosphate buffer system (PBS) and then incubated with the MAbs added in triplicate at concentrations ranging from 3.7x10^-11^ M to 75x10^-9^ M. After washing, the plates were incubated with human recombinant protein (HRP)-labeled goat anti-mouse immunoglobulin G [(IgG); BioRad] diluted 1:2000 in PBS for 1 h at RT. After washing, the enzymatic reaction was developed with 3,3',5,5'-tetramethylbenzidine (TMB) substrate (Sigma) and stopped by adding 1 M H_2_SO_4_. The optical density was measured at 450 nm (OD_450_) in a microtiter plate reader (Tecan). For each MAb concentration, the mean OD_450_ value (±SD) was calculated from triplicates. 

### Ethics statement

The use of human erythrocytes from healthy adult volunteer followed written informed consent was approved by the Council of the Institute of Biotechnology (Protocol of 30/03/2010, no. 3 with the extension for four years).

## Results

### Electrochemical impedance of tBLMs is affected by rVLY

**Figure 1:**
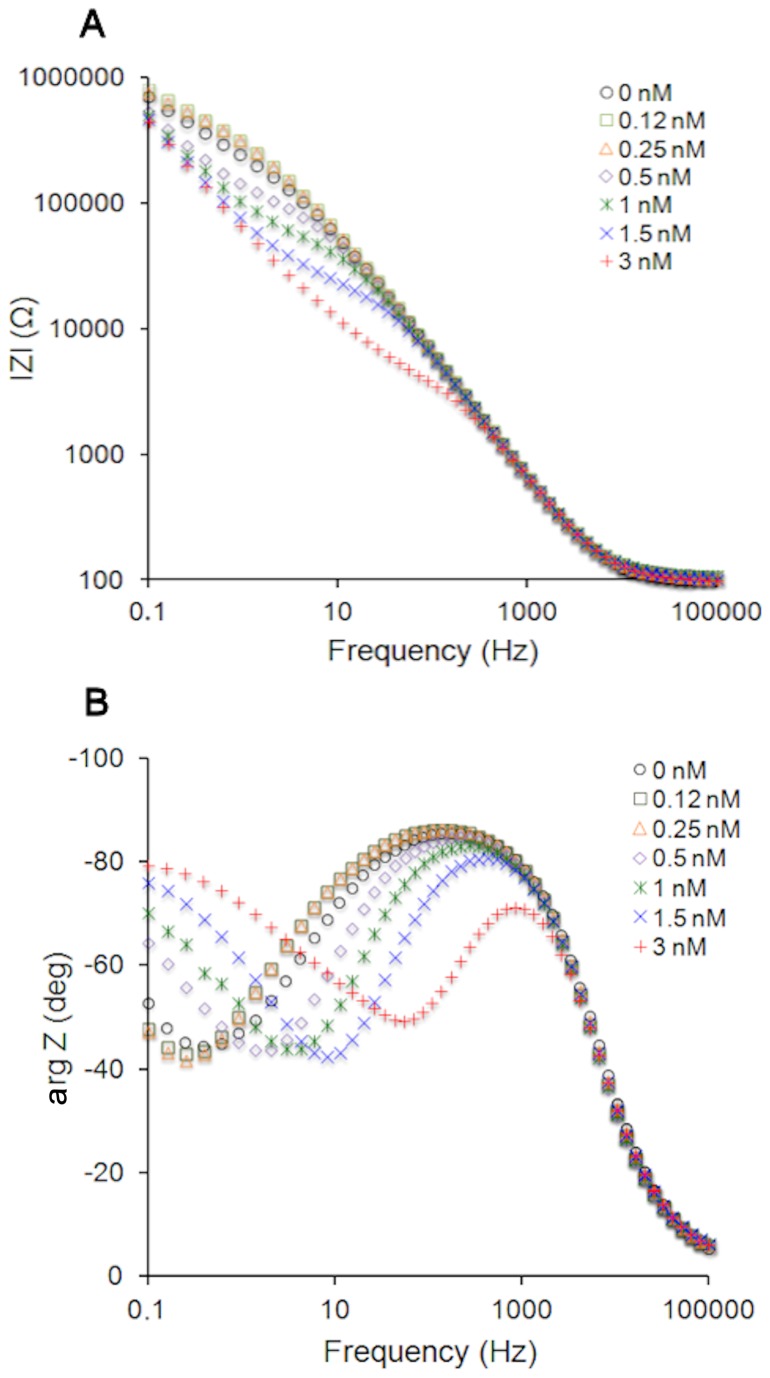
Impedance Bode plots of DOPC/CHOL40% tBLMs upon exposure to rVLY solutions of different concentrations. Exposure time 30 min. (A) Impedance magnitude. (B) Impedance phase.

Injection of rVLY into the buffer solution in contact with tBLMs triggers changes in the electrochemical impedance (EI) spectra of those tBLMs that contain cholesterol (Figure 1). In the absence of rVLY, Bode plots of the EI magnitude |Z| vs. frequency, exhibit an inflection centered around 0.2-0.3 Hz (Figure 1A), that coincides, on the frequency scale (Figure 1B), with the minimum of the negative of the impedance phase, - arg Z. Introduction of rVLY lowers |Z|, in the low frequency range, and triggers a shift of the phase minimum towards higher frequencies. Such features were observed for other pore-forming toxins such as αHL [20,34]. The extent of the EI spectral changes was found to be time dependent (data not shown). To elucidate the concentration dependent nature of the rVLY-membrane interaction, all curves in Figure 1 were recorded 30 min after injection. It is obvious that rVLY affects the EI spectra of the tBLMs in a concentration dependent manner. In control experiments, with no rVLY present, the tBLMs demonstrated EI parameter stability qualitatively and quantitatively. Additional control experiments using unrelated protein BSA in the concentration range from 10 to 100 nM indicated no effect on the experimental EI curves (see Figure S3 in File S2). Thus, the EI spectral changes seen in Figure 1 are unambiguously due to the presence of the added rVLY. Noteworthy, the EI spectral changes triggered by the addition of rVLY aliquots were irreversible, i.e. were not reversed by flushing the vials with rVLY-free buffer. Similar behavior is typically observed for β-barrel, pore-forming toxins [24,35,36].

To quantify the effect of rVLY on the electrical parameters of the tBLMs, as suggested by Cornell et al. [17,30,37,38], |Z| was measured at the frequency point, f_min_, at which the minimum of the negative of impedance phase is observed. Since the extent of membrane damage and defectiveness could be more conveniently related to the conductance of tBLMs at f_min_, hereinafter, the admittance, |Y_fmin_|= |Z_fmin_|^-1^, is used to characterize the interaction between rVLY and the tBLMs. More details on the methodology are presented in the Figure S1 in File S1 and File S1.

### rVLY-induced damage of tBLMs is dependent on cholesterol concentration

**Figure 2:**
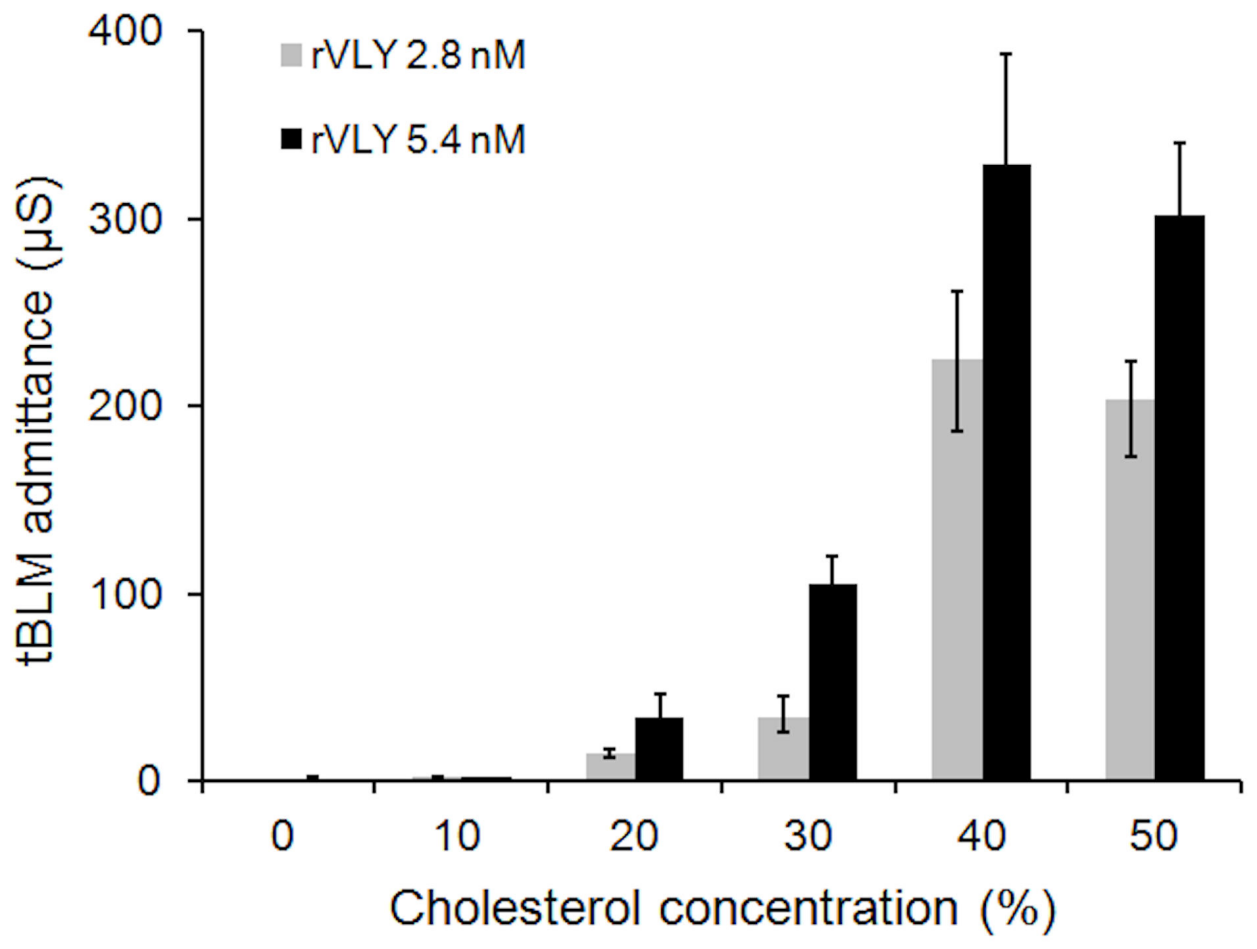
Cholesterol concentration-dependent tBLM admittance change triggered by rVLY. EIS recorded 30 min after the injection of rVLY. Incubation time 30 min.

Because rVLY belongs to a class of CDC, one may expect that the amount of cholesterol will affect the damage inflicted by rVLY to the membrane integrity. Such an effect is clearly demonstrated by the data in Figure 2. At two different protein concentrations (2.8 and 5.4 nM) the rVLY-induced admittance, |Y_fmin_|, depends on the cholesterol concentration in the DOPC tBLMs. At low cholesterol content (<10%), a marginal increase of the admittance of the tBLMs is observed. More than a 100-fold increase was detected at higher cholesterol concentrations (>20%) clearly indicating cholesterol is an essential component for activating the ability of rVLY to inflict damage to tBLMs, i.e. artificial phospholipid membranes. One possible cause for the inflicted damage is a cholesterol-induced change in the dielectric constant of the tBLM. It is well known that cholesterol lowers the dielectric constant within bilayer hydrophobic sheet [29]. To test for this, we modified the composition of the tBLMs using diphytanoylphosphocholine (DPhyPC) instead of DOPC. Although DPhyPC tBLMs exhibit significantly lower dielectric constants as compared to DOPC tBLMs [39], addition of rVLY resulted in no changes of the admittance of the cholesterol-free DPhyPC tBLMs (data not shown). These results eliminate dielectric constant factors as the cause the rVLY damage.

### Mutant rVLY variants with reduced hemolytic activity

To show that the observed effects presented in Figure 1 and Figure 2 are specific to rVLY and not related to some non-specific interaction of the peptide chain with the tBLM we used engineered mutant variants of rVLY with altered hemolytic activity (Figure S4). The rVLY mutants had amino acid (aa) substitutions in the VAARMQYD (aa 160-167) motif situated in the β1-strand of the VLY domain 3 (D3). Selection of the motif was based on previous publications reporting that the β1 and the β4 in D3 constitute the interface for monomer-monomer interaction in the perfringolysin oligomer [40]. The expression level in *E. coli* of each rVLY mutant was approximately in the same range as that of rVLY and the mutants were purified from the soluble fraction of *E. coli* lysate (data not shown). The hemolytic activity of the VLY mutants was determined using human erythrocytes. The aa substitutions altered the hemolytic activity of the rVLY mutants to a different extent (Table 1). The substitution of Ala-162 to the charged aa Glu (A162E) gave a non-hemolytic phenotype, while the substitutions Ala-162 to Val (A162V) and the Arg-163 to Val (R163V) decreased rVLY activity about 15 fold and four-fold, respectively. 

**Table 1:** Hemolytic activity of rVLY mutants.

**rVLY variants**	**HD_50_ (pM)**	**rVLY activity (%)**
rVLY	10±1	100
A162V	157±5	6
R163V	39±2	25
A162E	>2000	<0.01
T474G•L475G	>2000	<0.01

The double rVLY mutant was generated in which Thr-474 and Leu-475 were converted to glycines based on the previous report demonstrating an essential role of these aa residues for cholesterol binding [32]. The substitution of the Thr-Leu pair with Gly’s (T474G•L475G) resulted in a fully non-hemolytic rVLY phenotype (Table 1). In addition, the double mutant T474G•L475G did not show detectable binding to cholesterol-rich liposomes (data not shown).

To prove that the mutations did not alter the structure of rVLY, the activities of all generated rVLY mutants were tested with a panel of VLY-specific MAbs [13] and found to be reactive with the MAbs to the same extent as rVLY both by ELISA (Figure S5) and Western blot (data not shown). We also performed circular dichroism (CD) measurements of rVLY and the rVLY mutants. The CD spectra of the mutants were not discrepant from that of rVLY (Figure S6).

### Inactivated forms of rVLY mutants do not affect the electric properties of the tBLMs

**Figure 3:**
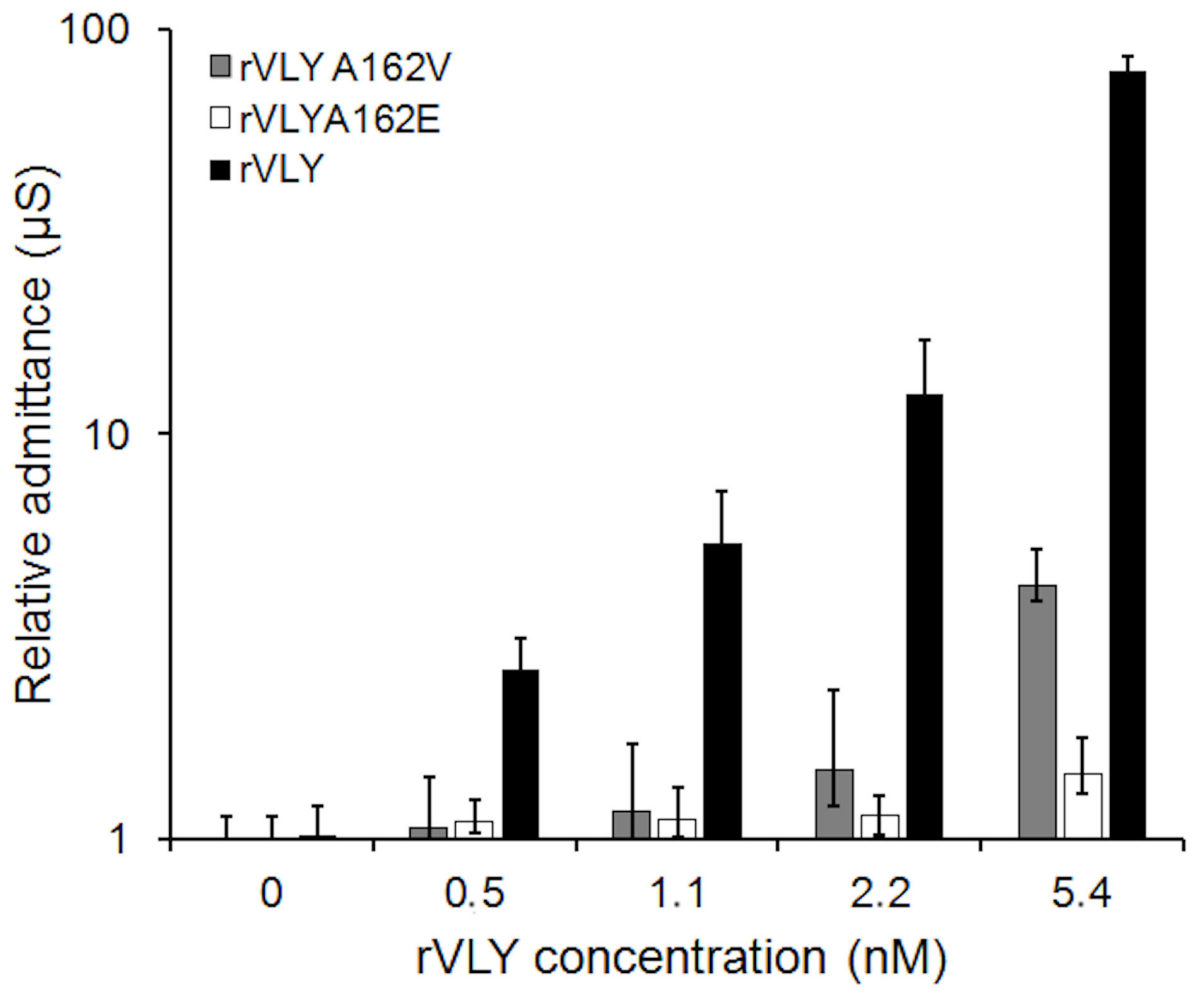
Admittance change of DOPC/CHOL40% tBLMs by different variants of VLY: rVLY (black bars), rVLY mutants A162E (white bars) and A162V (grey bars). Exposure time 30 min.

As the above data demonstrate, certain aa substitutions in the β1 strand of D3 almost fully inactivated hemolytic activity of rVLY (Table 1). To show whether the rVLY-tBLM interaction and the subsequent inflicted dielectric damage to the tBLMs correlate with the hemolytic activity of rVLY *in vitro*, we carried out parallel experiments with the rVLY mutants A162V, A162E and T474G•L475G on same-batch tBLMs. Data presented in Figure 3 show a significant, almost 70-fold increase in the admittance for the rVLY-tBLM interaction as compared to that for the partly inactive A165V mutant, in the same concentration range. Under the identical experimental conditions, the non-hemolytic mutants A162E (Figure 3) and T474G•L475G (data not shown) did not exhibit any effect on the insulating properties of the tBLMs.

### Neutralizing monoclonal antibodies against rVLY inhibit deterioration of electrical insulation of tBLMs

**Figure 4:**
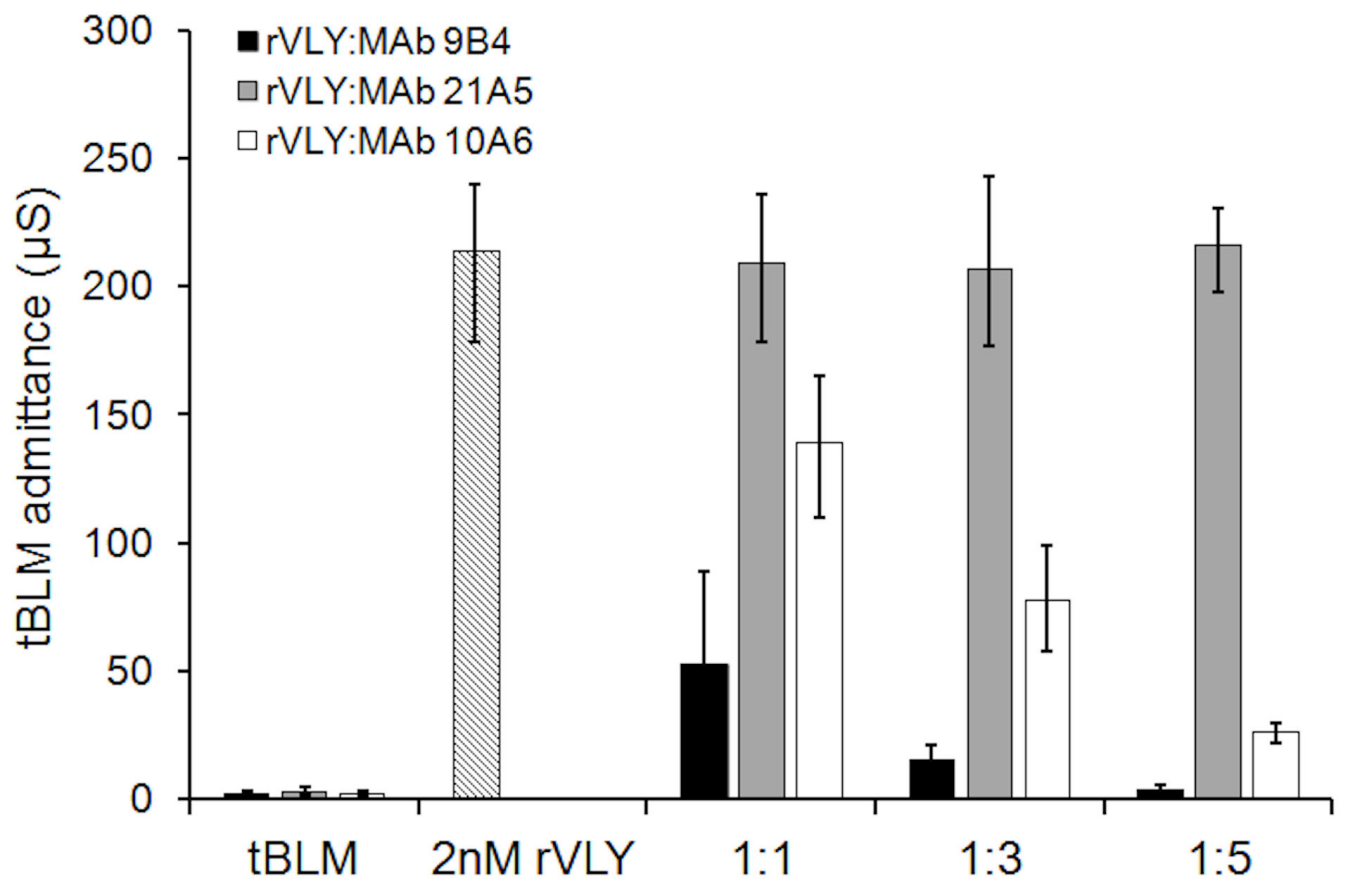
rVLY-induced admittance change of DOPC/CHOL 40% tBLMs after 30 min preincubation with different MAbs: neutralizing MAb 9B4 (black bars), moderate neutralizing MAb 10A6 (white bars) and non-neutralizing MAb 21A5 (grey bars). Exposure of the tBLMs to rVLY time 30 min. rVLY/MAb molar ratios indicated below the abscissa.

Neutralizing antibodies reduce the hemolytic activity of cytolysins. Recently, a series of monoclonal antibodies agaist rVLY with different inhibitory potencies was described [13]. We demonstrated that the neutralizing MAb 9B4 reduced the hemolytic activity of rVLYand its mutants in a dose-dependent manner while the non-neutralizing MAb 21A5 did not effect the hemolytic activity of rVLY and its derivatives (Figure S7). Neither MAb impacted the activity of non-hemolytic mutants A162E and T474G•L475G (Figure S7B). To test if preincubation of rVLY with the neutralizing MAb 9B4 affects its membrane damaging activity we carried out a series of experiments with different rVLY/MAb ratios (Figure 4). The control experiment included the preincubation of rVLY with non-neutralizing MAb 21A5. 

Clearly preincubation with the neutralizing MAb 9B4 inactivates the membrane-damaging activity of rVLY in a concentration-dependent manner. The non-neutralizing MAb 21A5 does not inhibit rVLY hemolytic activity *in vitro* [13] nor on the tBLM-damaging activity of rVLY.

### Tethered bilayer membranes act as real time sensors for VLY production in bacterial cultures

**Figure 5:**
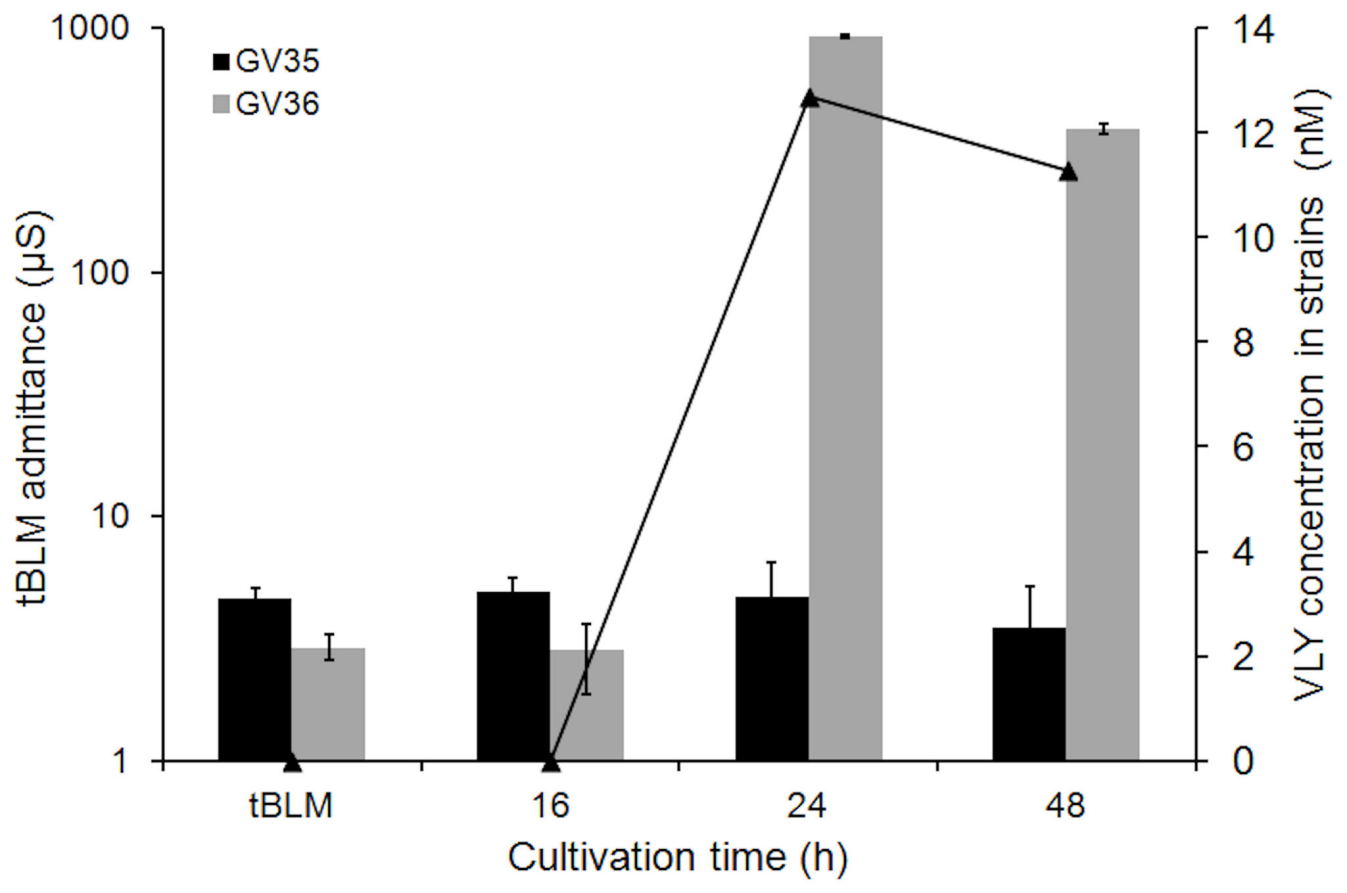
Quantification of VLY production in two *G. vaginalis* cultures by EIS and ELISA. Variation of the admittance of DOPC/CHOL40% tBLMs upon exposure to VLY in supernatants from two different strains of *G. vaginalis* (bars). VLY concentration (line) measured by ELISA. Exposure time of tBLM 30 min. Cultivation time of *G. vaginalis* is indicated under the abscissa.

To demonstrate the applicability of the tBLMs for sensing VLY in real biological media we investigated how culture supernatants from two strains of *G. vaginalis* affect the admittance. The VLY production levels of different *G. vaginalis* strains were determined by ELISA [14]. The bacterial supernatant from strain GV35 with undetectable level of VLY was found to be inactive towards the tBLMs (Figure 5). In contrast, the supernatant from the *G. vaginalis* strain GV36 with a high level of VLY exhibited a significant effect on the conductance of the tBLMs (Figure 5). Data presented in Figure 5 clearly demonstrate the correlation of an increase of membrane-damaging activity and growth time of bacterial culture, which is consistent with VLY accumulation in *G. vaginalis* cultures. VLY activity towards the tBLMs increases sharply (24 h) then decreases thereafter (48 hr). Thus, VLY-induced admittance of tBLM fully reproduces the VLY production pattern measured by ELISA. 

## Discussion

tBLMs may be utilized as bioanalytical devices for toxin sensing in cases where physical responses in a biologically relevant process are generated upon interaction with proteins [22]. Our data show that our rVLY preparations affect physical properties, in particular, the impairment of dielectric/insulating properties of tBLMs in a concentration-dependent manner (Figure 1). The decrease of the impedance magnitude (increase of admittance) and the shift of the negative of impedance phase minimum in the frequency range from 0.1 to 100 Hz (Figure 1), is most likely the result of the formation of a pore in the bilayer by the VLY oligomers, as predicted theoretically [19]. Nevertheless, variations similar to ones observed in Figure 1 may also be triggered by a non-specific interactions between peptide chains and the hydrophobic core of a bilayer. In other words, EIS features such as seen in Figure 1A and 1B may be observed without functional reconstitution of VLY and pore formation but simply due to protein adsorption to a membrane surface [41] or an insertion of protein that leads to an increase in the dielectric constant, a decrease in the dielectric barrier, and consequently in a loss of the ability of the phoholipid bilayer to prevent uncontrolable ion flow through the membrane [39]. For this reason we needed to establish that the changes triggered by the rVLY in tBLMs are biologically relevant. 

Biological relevance of the interaction between rVLY and the tBLMs could be verified by comparing the effects in tBLMs and hemolytic activity measured in cell culture. It is believed that pore formation in cell membrane is the primary reason for the cytotoxicity of VLY. Correlation of EIS features that are typical in the presence of water-filled defects with hemolytic activity would indirectly prove the existence of such pores and provide the biologically-sound basis for sensing and detection of this virulence factor by tBLM-based sensors. 

A straightforward way to test for biological relevance of protein-membrane interactions is to introduce mutations into the protein that would alter its biological activity (e.g. hemolytic activity) yet minimaly change the protein composition and structure, and, consequently, any presumed non-specific interaction between the protein and the membrane. Variation of the amino acid sequence in the rVLY region presumably involved in oligomerization [40] and, consequently, for the specific property of VLY to oligomerize into a pore strongly reduces the hemolytic activity of rVLY for the A162E and T474G•L475G substitutions (Table 1). The EIS spectra of the tBLMs exposed to solutions (≈1 nM) of these mutants indicated no activity (data not shown), in agreement with the *in vitro* hemolytic activity data. The A162V and R163V substitutions supress hemolytic activity only partly (Table 1). Again, the EIS data follows the same pattern. The admittance increase is less in the A162V mutant compared to that for rVLY. At lower rVLY concentrations (<1.1 nM) the hemolytic activity decrease and the admittance increase match almost quantitatively, i.e. for the A162V mutant (HD_50_ almost 16-fold higher while the toxin-induced admittance change is ≈10-15 times lower). At higher concentrations, the A162V-induced admitance chanel is 3 to 10 times less prononced relative to rVLY (Figure 3, compare black and grey bars). The data with the mutants suggest that membrane changes induced by rVLY match the biological activity of the toxin, strongly supporting the hypothesis that the EIS variations indicate protein insertion and pore-formation in the artificial membrane. Importantly, the experiments with the rVLY mutants not only prove the biological relevance of the tBLM EIS measurements they also confirm the ability of rVLY and its partially inactivated variants to bind and form protein pores in membranes in the abscence of CD59. 

Another confirmation of the biological relevance of a tBLM response to VLY can be obtained using neutralizing and non-neutralizing monoclonal antibodies (Figure 4). As expected, MAb 9B4, which fully neutralizes the cytolytic activity of rVLY [13] removed any effect of rVLY on the tBLM insulating properties. The action of the MAb 9B4 is concentration-dependent (Figure 4) proving that antibody binding to a rVLY is inhibiting electrochemical changes in the tBLMs. Within the margin of error, no changes in the tBLM admittance can be detected if rVLY is preincubated with MAb 9B4 at the protein/antibody ratio larger than 1:5. Another antibody MAb 10A6, which exhibits moderate neutralizing activity in hemolytic tests [13] inhibited the tBLM dielectric damage to a lesser extent (Figure 4). Finally, pre-incubation of rVLY with non-neutralizing MAb 21A5 showed no inhibitory effect in the membrane admittance change (Figure 4). Taken together, the neutralization experiments rule out the possibility of rVLY-tBLM non-specific interactions as a cause of the EIS changes (Figure 1).

Recent studies show that the amount of VLY produced by different *G. vaginalis* clinical isolates cultivated *in vitro* can be detected by immunoassays based on either polyclonal or monoclonal VLY-specific antibodies [14,16] and that *G. vaginalis* strains differed in VLY production levels [14]. Correlation of the level of produced toxin with electrochemical response would also confirm that the VLY-tBLM interactions are biologically relevant as well as demonstrate the applicability of the tBLM sensors for monitoring toxin production in media.

The amount of VLY produced by different *G. vaginalis* strains and the EIS response obtained on the tBLMs are completely analogous (Figure 5). With the bacterial strain GV35, which did not produce detectable amounts of VLY [14], no variation of the EIS and the admittance of the tBLM was observed. A different EIS response was observed with the supernatants from GV36 strain that demonstrated production of significant amouts of VLY, up to 10-12 nM as determined by ELISA (Figure 5, line curve) and the admittance of the cholesterol-containing tBLMs closely followed the ELISA data. Interestingly, even such subtle details as the slight decrease of VLY concentration in the media after 24 hrs of cultivation, is reproduced by the variation of the admittance of tBLMs proving the applicability of the tBLM sensors in bioanalytical applications to detect both the activity and amout of produced VLY in real media.

Our data strongly suggests that VLY is capable of membrane binding and forming pore-like defects in the absence of the human CD59 receptor. This finding is quite unexpected because there exists evidence to the contrary [6,25] and forms the basis of the conclusion that CD59 is a principle mediator of VLY pore-formation and toxicity, along with cholesterol. We show uniquivically that the binding of VLY to a bilayer occurs without mediation of human CD59, while the requirement for the cholesterol is strictly unconditional. We performed some numerical estimates of the density of defects (presumably, pores) produced by VLY in the tBLMs, following the EIS formalism developed earlier [19] to explain this seeming contradiction with the biological data [6,14].

The defect density in pristine tBLMs estimated from the EIS Bode spectra is less than 0.01 µm^-2^ [phase minimum at f_min_≈0.4 Hz (Figure 1B ); see formula eq. (34) in ref. 17]. However, the accuracy of such estimates are dependent on knowing the defect radius. Because of a lack of structural data on VLY we can make only a rough estimate of the maximum density of protein pores. Favourably, the theory predicts a weak dependence of the position of -arg Z vs. frequency on defect size [19]. Assuming the radius of the VLY pore falls into a rather wide interval from 1 to 10 nm, we obtain maximal densities of defects that are summarized in Table 2. Maximal densities are obtained assuming a pore radius of 1 nm and increasing the pore radius to 10 nm results in a decrease of the upper limits by only 1.58. These theoretical estimates show that, even in the absence of CD59, VLY is capable of producing significant pore densities in tBLMs (Table 2). However, one needs to take into account that the concentrations at which the EIS experiments were carried out are well above the physiological concentrations of CDCs in natural media (see e.g. ref [42]). For human erythrocytes that contain CD59 the HD_50_ for rVLY is observed at 0.01 nM (Table 1), which is at least a factor of 50 lower than any reliably detectable activity of rVLY, as measured in this work on CD59-free tBLMs (at 0.5 nM rVLY). This suggests that in the absence of CD59 relatively large concentrations of VLY are required for reconstitution into functional oligomeric pores, in good agreement with earlier rVLY toxicity tests obtained utilizing mouse erythrocytes that lack human CD59. The HD_50_ for mouse erythrocytes is approximately 200-fold higher than that for human erythrocytes [23]. As a result we conclude that both the biological toxicity data and electrochemical data obtained in this study are in good qualitative and quantitative agreement and confirm functional reconstitution of VLY into model bilayer membranes.

**Table 2:** Estimates of defect density and number of pores per model erythrocyte.

**rVLY concentration (nM)**	**f_min_ (Hz)**	**Density of pores in tBLM (µm^-2^)**	**Number of pores per model erythrocyte**
0	0.4	<0.02	n/a
0.12	0.3	<0.01	<1
0.25	0.3	<0.01	<1
0.5	1.4	<0.28	<22
1	4.2	<2	<180
1.5	8.1	<8	<650
3	55	<330	<2.6⋅10^4^

Assuming a model erythrocyte has the shape of an oblate spheroid we can estimate the number of VLY-generated defects. With an equatorial and an axial radius of a =3.5 µm and c =1 µm, respectively, the surface area of such a hypothetical erythrocyte is estimated to be ≈80 µm^2^ by the following equation [43]:

### $S=2\pi a^{2}+\pi \frac{c^{2}}{e}\ln\left(\frac{1+e}{1-e}\right)$

where the ellipticity parameter is defined as:

$e=\sqrt{1-\frac{c^{2}}{a^{2}}}$ .

Taking into account the defect density data (Table 2, third column) one may estimate the upper defect limit present on the surface of hypothetical erythrocyte at a particular VLY concentration (Table 2, fourth column). At low rVLY concentrations, e.g. 0.25 nM (14 ng/mL), the number of defects per erythrocyte is well below 1 and no hemolytic activity is detected in mouse erythrocyte tests [23]. However, at 1 nM VLY (≈60 ng/mL) almost 50% of mouse erythrocytes are lysed. The EIS data in such a situation show up to 180 defects present in the membrane for the above hypothetical erythrocyte. This strongly suggests that in order to exert noticeable hemolytic activity at least 100-200 toxin pores must form on the surface of a cell. 

Interestingly, our data shows that cholesterol plays a decisive role in VLY pore formation. The absence of VLY effects on the DPhyPC tBLMs indicates that the receptor type of cholesterol-VLY interaction is responsible for VLY hemolytic activity, not lowering of the dielectric constant as for αHL [29]. The absolute requirement for cholesterol is apparent from the fact that even at rVLY concentrations as high as 5 nM (280 ng/mL, Figure 2) membrane damaging activity can be essentially nullified by decreasing the tBLM cholesterol content to 10%. We show in this work that CD59 is not an essential factor for VLY hemolytic activity; however, it probably strongly amplifies toxicity by either mediating monomer binding to the phospholipid membrane or by accelerating membrane-mediated oligomerization into the pore [44]. 

## Conclusions

Artificial membranes tethered to a surface are biologically relevant models for studying interactions between CDCs, in particular, VLY with phospholipid membranes. They are suitable and may be utilized as sensors for the detection of the activity of VLY, and possibly other pore-forming CDCs. Currently the sensitivity of our tBLM sensor, which is determined solely by the residual specific admittance of the membrane, is close to 0.5 nM (≈28 ng/mL). This concentration is approximately 3 orders of a magnitude lower than that achieved by optical techniques utilizing tBLMs (see e.g. surface plasmon resonance spectroscopy methodology for the label-free detection of cholera toxin [45]) and is comparable to the sensitivity of amperometric electrochemical detection of streptolysin from *Streptococcus pyogenes* [46,47]. The sensitivity achieved in this work slightly surpasses that of current ELISA methodologies, capable of detecting VLY down to ≈100 ng/mL [14]. The limited data on the physiologic concentrations of pore-forming toxins currently does not allow the conclusion that the sensitivity achieved in this work is sufficient to detect VLY in biological specimens such as vaginal smears. Further development of tBLMs suitable for applications in real clinical samples is in progress. Nevertheless, in biotechnology and other indirect diagnostic applications, for example, in cultivation media of pathogenic bacteria, the levels of VLY could reach higher concentrations. The current work demonstrates that data obtained using tBLM sensors match well to that obtained using classical ELISA technology indicating the tBLMs may be applicable for fast, automated, real time detection of this toxin in cultivation media. The current study provides unequivocal proof for the membrane-binding and the pore-forming ability of VLY into a phospholipid membrane in the absence of the human complement protein CD59. While it is not clear what the molecular mechanism of the CD59 activity is – increasing the amount of membrane bound protein or accelerating oligomerization on the membrane surface – it is evident that the introduction of CD59 into an artificial tBLM architecture would appear to be a promising way to further sensitize the detection of this and similar pathogens.

## Supporting Information

File S1 **EIS methodology for measuring membrane damage by the pore forming toxins.** **File contains the following figures: Figure S1**. (A) The electrical equivalent circuit of an idealized phospholipid bilayer surrounded from two sides by a conducting medium (electrolyte solution). R(sol) represents the resistance of an electrolyte solution, C(mem) represents the capacitance of a phospholipid bilayer membrane. (B) The electrical equivalent circuit of a bilayer membrane subject to a pore-forming toxin, which creates an additional conductance pathway modeled by the impedance Z(defect). **Figure S2**. Model Bode plots of the electrochemical impedance spectra. (A) Impedance magnitude, and (B) impedance phase vs. frequency curves. Blue curves represent the impedance of an ideal, defect-free bilayer. Red and green curves represent the impedance curves of the membranes containing small (red) and large (green) number of defects. Parameters for model curves are, as follows: R(sol) = 100 Ω, C(mem) = 0.3 µF. Z(defect) was modeled by a series RC element, which had the following values R = 10^6^ Ω and C=3·10^-6^ µF (red curves); R=10^4^ Ω and C=3·10^-6^ µF (green curves).
(PDF)

File S2 **Membrane damage: negative control with the unrelated bovine serum albumin protein.** **File contains the Figure S3**. Impedance Bode plots of DOPC/CHOL 40% tBLMs (black circles) upon exposure to 100 nM BSA solution (red triangles). Exposure time 30 min. (A) Impedance magnitude, (B) Impedance phase.
(PDF)

Figure S4 **Schematic representation of the VLY structure and the positions of the aa mutations.** The model of full-length VLY domain structure is based on the homology with ILY. Black area indicates the binding site for the neutralizing MAb 9B4 [Ref. 13 in the main article]. rVLY lacked the putative signal sequence (1-31 aa) (dashed area).
(TIF)

Figure S5 **Binding of the MAbs 9B4 (**A**) and 21A5 (**B**) to rVLY and its mutants determined by an indirect ELISA.** The MAbs were incubated at concentrations ranging from 3.7x10^-11^ M to 46x10^-9^ M on the microtiter plates coated with the respective antigens. For each MAb concentration, the mean OD_450_ values (+SD) calculated from triplicates are indicated. Error bars represent 95% confidence intervals (CI) of mean value where indicated.
(TIF)

Figure S6 **Circular dichroism (CD) spectra of rVLY and its mutants in 20 mM sodium acetate buffer pH 5.5.** MRE – mean residue ellipticity.
(TIF)

Figure S7 **The effect of the MAbs 9B4 and 21A5 on the hemolytic activity of rVLY and its mutant variants.** The effect of MAbs 9B4 and 21A5 on the hemolytic activity of rVLY and rVLY mutant variants was tested by addition of human erythrocyte suspension to (A) rVLY (5 ng/mL) pre-incubated with either the neutralizing MAb 9B4 [Pleckaityte et al., 2011] or non-neutralizing MAb 21A5 at concentrations ranging from 6.7x10^-11^ to 0.4x10^-9^ M; rVLY mutant R163V (10 ng/mL) pre-incubated with either 9B4 or 21A5 MAb at concentrations ranging from 6.7x10^-11^ M to 0.4x10^-9^ M; (B) rVLY mutant A162V (30 ng/mL) pre-incubated with either 9B4 or 21A5 MAb at concentrations ranging from 1x10^-9^ M to 6x10^-9^M; rVLY mutant A162E (750 ng/mL) pre-incubated with either 9B4 or 21A5 MAb at concentrations ranging from 1x10^-9^ M to 7x10^-9^ M. Error bars represent 95% CIs of mean value where indicated.
(TIF)
