# Supplementary material for: Reconstitution of Cholesterol-Dependent Vaginolysin into Tethered Phospholipid Bilayers: Implications for Bioanalysis
Source: PLoS One. 2013 Dec 13;8(12):e82536. doi: 10.1371/journal.pone.0082536 (PMC3862629; doi:10.1371/journal.pone.0082536)
Supplement: File S1 — EIS methodology for measuring membrane damage by the pore forming toxins. File contains the following figures: Figure S1. (A) The electrical equivalent circuit of an idealized phospholipid bilayer surrounded from two sides by a conducting medium (electrolyte solution). R(sol) represents the resistance of an electrolyte solution, C(mem) represents the capacitance of a phospholipid bilayer membrane. (B) The electrical equivalent circuit of a bilayer membrane subject to a pore-forming toxin, which creates an additional conductance pathway modeled by the impedance Z(defect). Figure S2. Model Bode plots of the electrochemical impedance spectra. (A) Impedance magnitude, and (B) impedance phase vs. frequency curves. Blue curves represent the impedance of an ideal, defect-free bilayer. Red and green curves represent the impedance curves of the membranes containing small (red) and large (green) number of defects. Parameters for model curves are, as follows: R(sol) = 100 Ω, C(mem) = 0.3 µF. Z(defect) was modeled by a series RC element, which had the following values R = 106 Ω and C=3·10-6 µF (red curves); R=104 Ω and C=3·10-6 µF (green curves). (PDF) [file pone.0082536.s001.pdf]

Supporting information for "Reconstitution of cholesterol-dependent vaginolysin into tethered phospholipid bilayers: implications for bioanalysis".

Rima Budvytyte, Milda Pleckaityte, Aurelija Zvirbliene, David J. Vanderah, Gintaras Valincius

EIS methodology for measuring membrane damage by the pore forming toxins

In an alternating electric field, the ideal defect-free membranes act as a perfect insulating sheet surrounded from two sides by a conducting media. Such arrangement of conductors/insulators may be represented by an electric equivalent circuit shown in Fig. S1A. The EI spectra of such a circuit in the so-called Bode representation are shown in Fig S2 (blue curves).

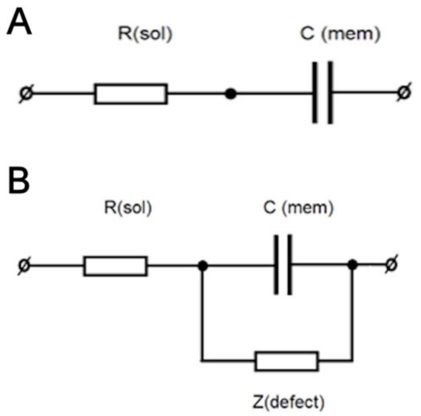

Figure S1. (A) The electrical equivalent circuit of an idealized phospholipid bilayer surrounded from two sides by a conducting medium (electrolyte solution). R(sol) represents the resistance of an electrolyte solution, C(mem) represents the capacitance of a phospholipid bilayer membrane. (B) The electrical equivalent circuit of a bilayer membrane subject to a pore-forming toxin, which creates an additional conductance pathway modeled by the impedance Z(defect).

Real membranes, in contrast to the ideal ones, contain defects. Those defects create additional conductance pathways in a membrane. Such pathways must be modeled by a parallel conductance element, which in our case is denoted by Z(defect). Detailed analysis of the Z(defect) impedance is presented in ref. [1]. In general, Z(defect) is a complex function of parameters of the tethered bilayer membranes. However, measured impedance spectra contain some features that can be directly related to a presence of defects in the membranes.

An impedance plot of a defect-free ideal membrane, calculated using equivalent model in Fig. S1A is shown in Fig. S2. The impedance magnitude curve below $f \approx 10^4$ Hz exhibits linear behavior, while the impedance phase slowly approaches -90 degrees (Fig. S2, blue curves). The high frequency flat spectral range (above $f \approx 10^4$ Hz) corresponds to a situation, in which the measured impedance is totally determined by the solution resistance. Consequently, this spectral range contains no physical information about processes in the bilayers.

The presence of defects in real-membranes is manifested by the appearance of step-like inflections on the impedance magnitude curve and a minimum of a negative of the impedance phase curve (Fig. S2, red and green curves). The height and the position of the step are determined by the density of the membrane defects. The midpoint of the step coincides with the position of the extremum on the phase plot (Fig. S2B).

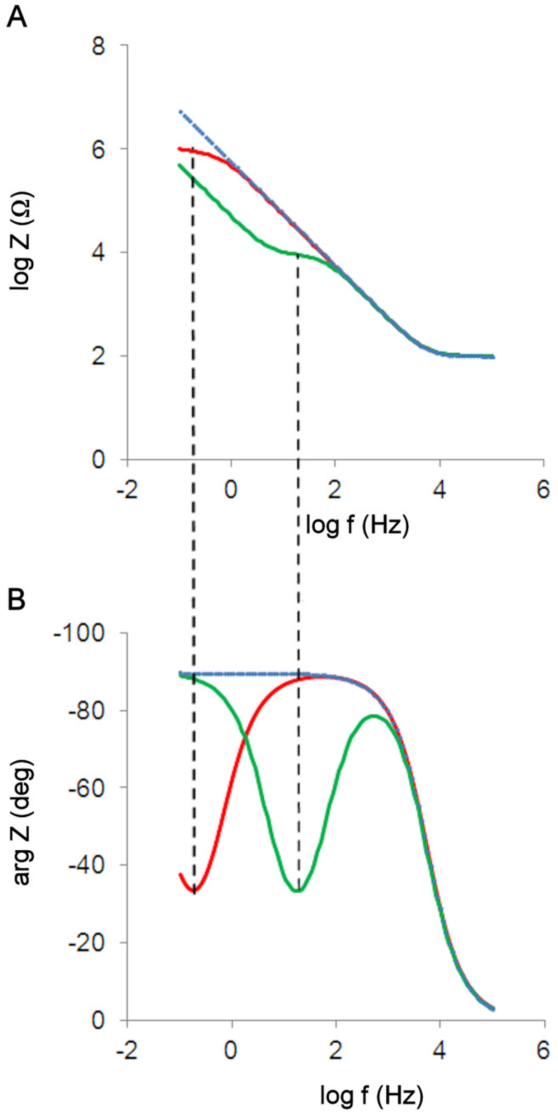

Figure S2. Model Bode plots of the electrochemical impedance spectra. (A) Impedance magnitude, and (B) impedance phase vs. frequency curves. Blue curves represent the impedance of an ideal, defect-free bilayer. Red and green curves represent the impedance curves of the membranes containing small (red) and large (green) number of defects. Parameters for model curves are, as follows: R(sol) = 100 Ω, C(mem) = 0.3 μF. Z(defect) was modeled by a series RC element, which had the following values R = 10^6 Ω and C=$3\cdot10^{-6}$ μF (red curves); R=10^4 Ω and C=$3\cdot10^{-6}$ μF (green curves).

In our case, when the bilayer membrane is exposed to a media containing rVLY, the pore-forming toxin reconstitutes into the membrane producing additional water-filled conductance pathways. Electrically, this is equivalent to generating more defects in addition to those already in the membrane. The position of the step-like feature in the Bode plot reflects the activity of this pore-forming toxin, which is observed in our work (see Fig. 1 in the article).

Analysis carried out in ref. [1] indicates that the impedance magnitude at f_{min} correlates with the defect density as $Z_{fmin} \sim 1/N_{def}$ (N_{def} is the defect density), so the admittance $Y_{fmin} = Z_{fmin}^{-1}$ at f_{min} is a near linear function of this toxin-induced defect density.[1] Consequently, Y_{min} can be used to quantify the activity of a pore-forming toxin. In the model spectra (Fig. S2) the curves depict three situations: no defects, Y_{min}=0 (blue); small defect density Y_{min}= 1 µS (red), and large defect density Y_{min}= 100 µS (green).

References:

1. Valincius G, Meskauskas T, Ivanauskas F (2012) Electrochemical impedance spectroscopy of tethered bilayer membranes. Langmuir 28 :977-990.

[1] Analysis in ref S1 shows that the functional relationship between Z_{fmin} and $1/N_{def}$ is nearly linear and follows a Z_{fmin} = const*$(1/N_{def})^{1.06}$ law.
