# Supplementary material for: Reconstitution of Cholesterol-Dependent Vaginolysin into Tethered Phospholipid Bilayers: Implications for Bioanalysis
Source: PLoS One. 2013 Dec 13;8(12):e82536. doi: 10.1371/journal.pone.0082536 (PMC3862629; doi:10.1371/journal.pone.0082536)
Supplement: File S2 — Membrane damage: negative control with the unrelated bovine serum albumin protein. File contains the Figure S3. Impedance Bode plots of DOPC/CHOL 40% tBLMs (black circles) upon exposure to 100 nM BSA solution (red triangles). Exposure time 30 min. (A) Impedance magnitude, (B) Impedance phase. (PDF) [file pone.0082536.s002.pdf]

Supporting information for "Reconstitution of cholesterol-dependent vaginolysin into tethered phospholipid bilayers: implications for bioanalysis".

Rima Budvytyte, Milda Pleckaityte, Aurelija Zvirbliene, David J. Vanderah, Gintaras Valincius

Membrane damage: negative control with the unrelated bovine serum albumin protein

tBLMs, like natural biological membranes, are highly resistant to non-specific adsorption of proteins. However, to ensure there is no effect on electrochemical impedance data from non-specific adsorption, we tested the tBLMs using the non-related protein, bovine serum albumin (BSA). BSA is the most abundant blood plasma protein, with a molecular weight of 66 kDa, similar to that of rVLY (~56 kDa), and is frequently used in tests for non-specific adsorption [1]. We tested the effect of BSA up to a concentration of 100 nM, which is more than 20 times the concentrations of VLY protein used in this work.

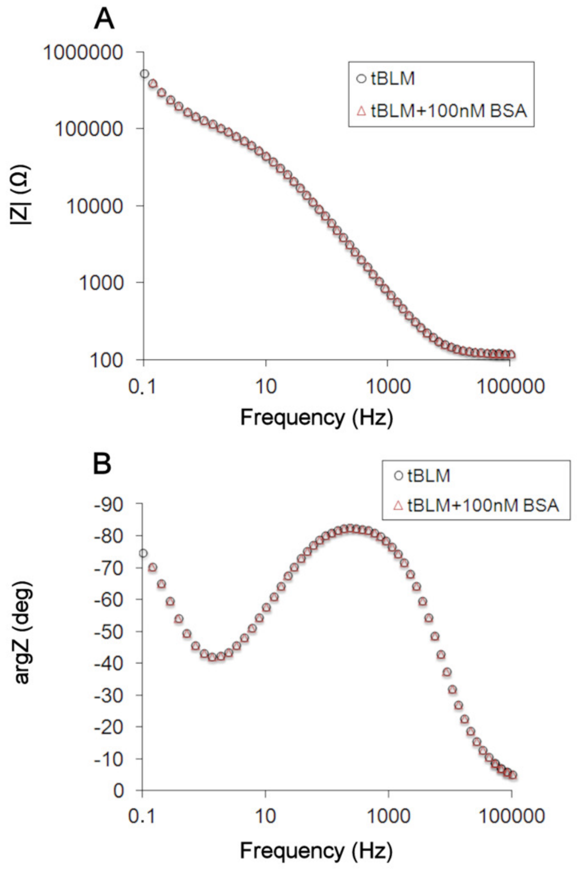

Figure S3. Impedance Bode plots of DOPC/CHOL 40% tBLMs (black circles) upon exposure to 100 nM BSA solution (red triangles). Exposure time 30 min. (A) Impedance magnitude, (B) Impedance phase.

Figure S3 displays Bode plots of the electrochemical impedance spectra before and after the addition of 100 nM BSA. No significant effect on the electrochemical impedance spectra was detected. Consequently, in the current study, the non- specific binding of proteins was below the detection limit of the method.

References:

1. Vanderah DJ, La H, Naff J, Silin V, Rubinson KA (2004) Control of protein adsorption: molecular level structural and spatial variables. JACS 126: 13639-13641.
