# Supplementary figures and images for: Reconstitution of Cholesterol-Dependent Vaginolysin into Tethered Phospholipid Bilayers: Implications for Bioanalysis
Source: PLoS One. 2013 Dec 13;8(12):e82536. doi: 10.1371/journal.pone.0082536 (PMC3862629; doi:10.1371/journal.pone.0082536)

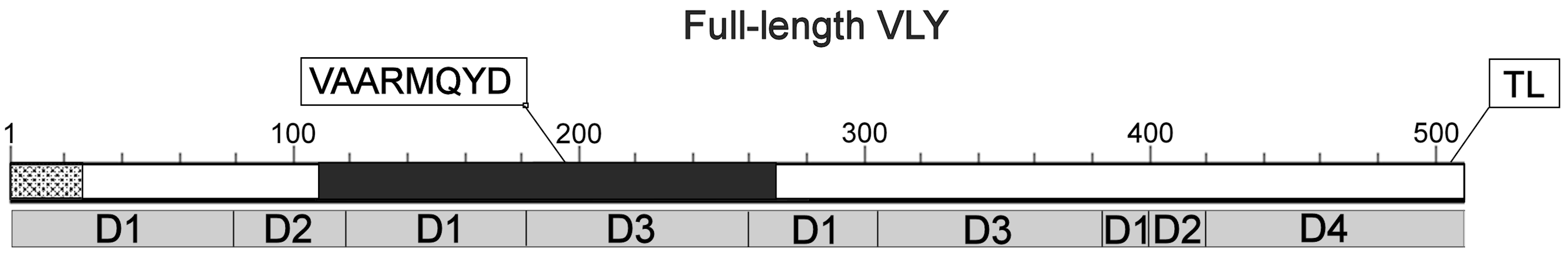

Supplement: Figure S4 — Schematic representation of the VLY structure and the positions of the aa mutations. The model of full-length VLY domain structure is based on the homology with ILY. Black area indicates the binding site for the neutralizing MAb 9B4 [Ref. 13 in the main article]. rVLY lacked the putative signal sequence (1-31 aa) (dashed area). (TIF) [file pone.0082536.s003.tif]

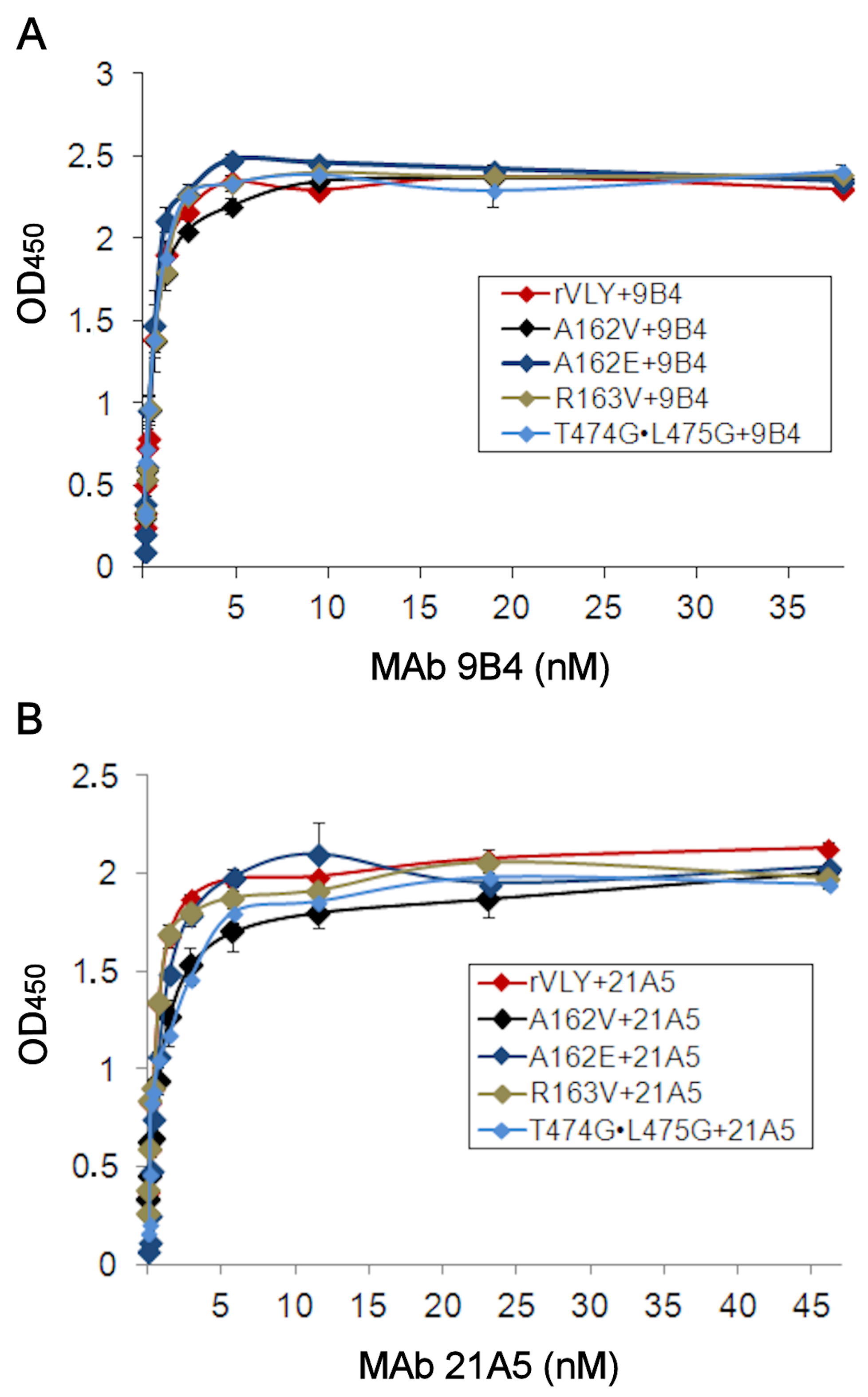

Supplement: Figure S5 — Binding of the MAbs 9B4 (A) and 21A5 (B) to rVLY and its mutants determined by an indirect ELISA. The MAbs were incubated at concentrations ranging from 3.7x10^-11^ M to 46x10-9 M on the microtiter plates coated with the respective antigens. For each MAb concentration, the mean OD450 values (+SD) calculated from triplicates are indicated. Error bars represent 95% confidence intervals (CI) of mean value where indicated. (TIF) [file pone.0082536.s004.tif]

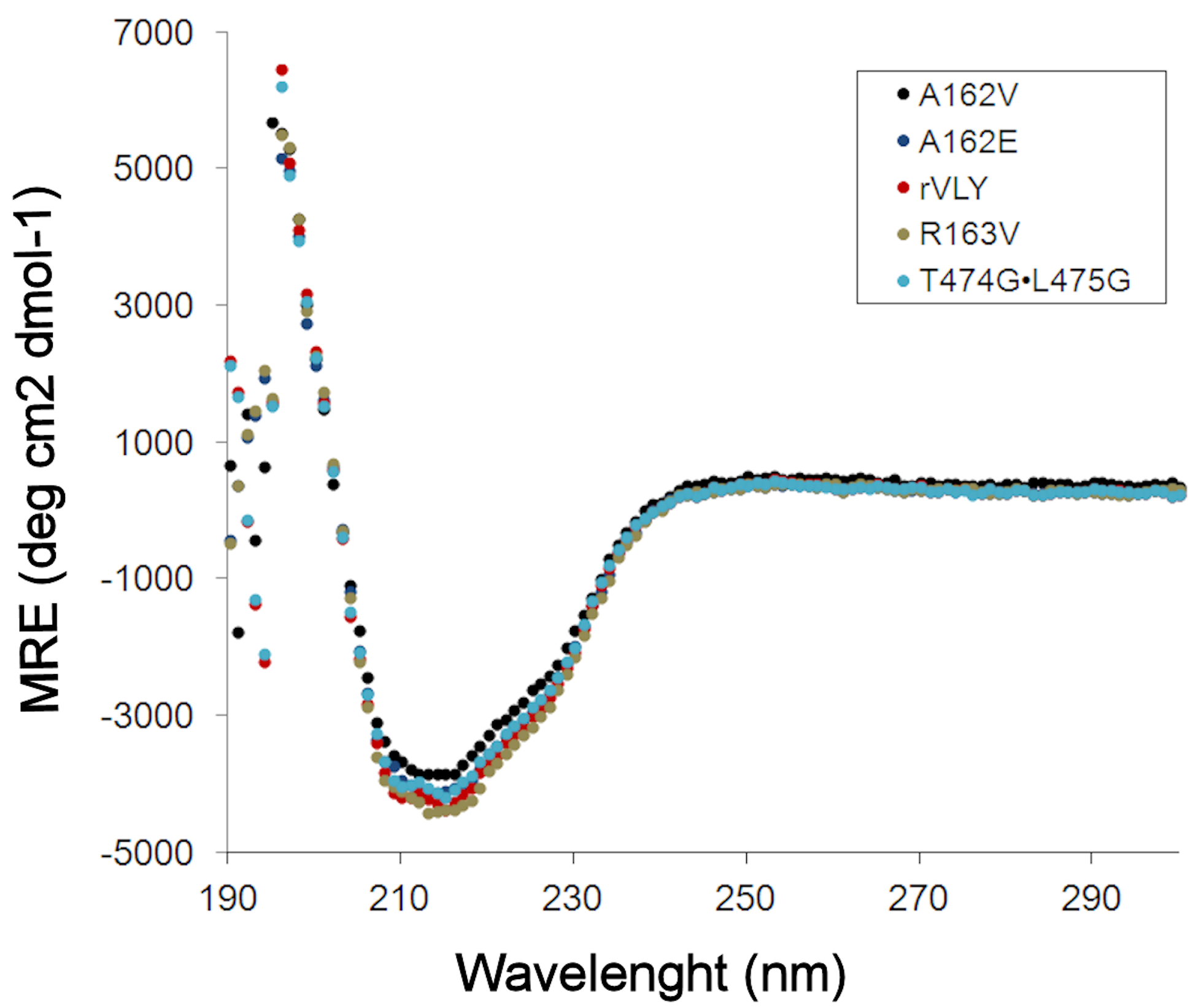

Supplement: Figure S6 — Circular dichroism (CD) spectra of rVLY and its mutants in 20 mM sodium acetate buffer pH 5.5. MRE – mean residue ellipticity. (TIF) [file pone.0082536.s005.tif]

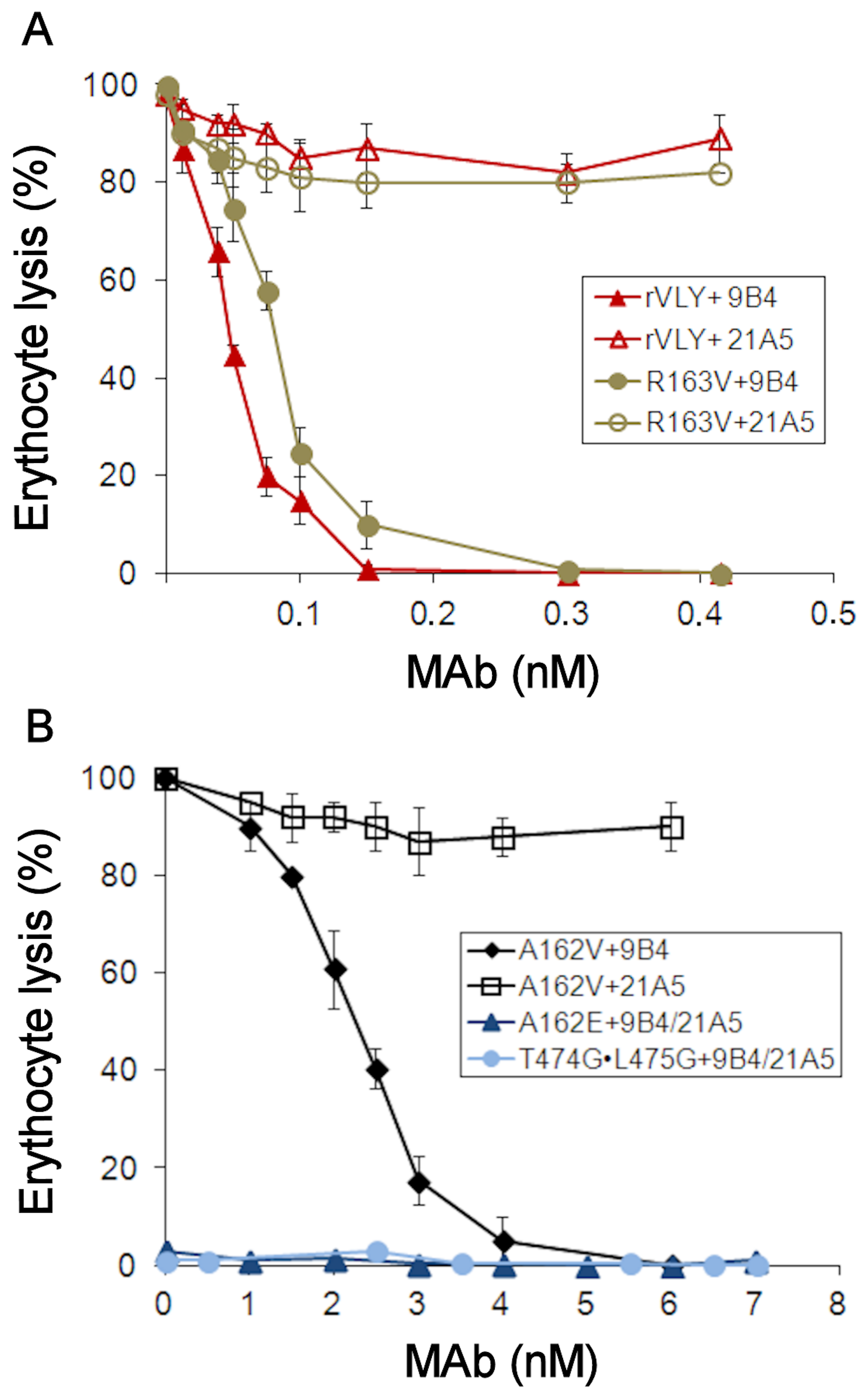

Supplement: Figure S7 — The effect of the MAbs 9B4 and 21A5 on the hemolytic activity of rVLY and its mutant variants. The effect of MAbs 9B4 and 21A5 on the hemolytic activity of rVLY and rVLY mutant variants was tested by addition of human erythrocyte suspension to (A) rVLY (5 ng/mL) pre-incubated with either the neutralizing MAb 9B4 [Pleckaityte et al., 2011] or non-neutralizing MAb 21A5 at concentrations ranging from 6.7x10^-11^ to 0.4x10^-9^ M; rVLY mutant R163V (10 ng/mL) pre-incubated with either 9B4 or 21A5 MAb at concentrations ranging from 6.7x10^-11^ M to 0.4x10^-9^ M; (B) rVLY mutant A162V (30 ng/mL) pre-incubated with either 9B4 or 21A5 MAb at concentrations ranging from 1x10^-9^ M to 6x10^-9^M; rVLY mutant A162E (750 ng/mL) pre-incubated with either 9B4 or 21A5 MAb at concentrations ranging from 1x10^-9^ M to 7x10^-9^ M. Error bars represent 95% CIs of mean value where indicated. (TIF) [file pone.0082536.s006.tif]
